# Triple Total Coronary Occlusions in a Young Adult: Looking Beyond Conventional Atherosclerotic Risk Factors

**DOI:** 10.7759/cureus.119030

**Published:** 2026-11-02

**Authors:** Junaid Naeem, Naseema-Maria Begum, Faraz Amjad

**Affiliations:** 1 Hospital Medicine, Manchester University NHS Foundation Trust, Manchester, GBR; 2 General Internal Medicine, Manchester University NHS Foundation Trust, Manchester, GBR; 3 Cardiology, Manchester University NHS Foundation Trust, Manchester, GBR

**Keywords:** acute myocardial infarction, electronic-cigarette, kawasaki disease (kd), premature coronary artery disease, total coronary occlusion

## Abstract

Severe coronary artery disease is uncommon in young adults, particularly when conventional cardiovascular risk factors are limited. We describe a 26-year-old man who presented with exertional chest pain and was found to have total occlusions affecting all three major coronary arteries. Extensive investigation for inherited, metabolic, autoimmune, and inflammatory causes did not identify a definitive aetiology. His history of heavy electronic-cigarette use and a prolonged febrile illness in childhood were considered potentially relevant but could not be established as causal. He ultimately underwent successful three-vessel coronary artery bypass grafting. This case highlights the importance of investigating unusual causes of severe premature coronary disease while avoiding over-attribution to unconfirmed risk factors.

## Introduction

Acute myocardial infarction (AMI) and obstructive coronary artery disease (CAD) are uncommon in very young adults, and the angiographic pattern in younger patients may differ from that observed in older populations [1,2]. Cigarette smoking, dyslipidaemia and a family history of premature CAD are important risk factors in younger patients; however, the identification of severe coronary disease in a very young patient with limited conventional cardiovascular risk factors presents a diagnostic challenge [1,2]. A total coronary occlusion represents complete obstruction of a coronary artery, with distal myocardial perfusion potentially dependent on collateral circulation when the occlusion is longstanding.

Potential explanations include inherited disorders of lipid metabolism, inflammatory or autoimmune arteriopathy, recreational drug exposure, congenital coronary abnormalities, and sequelae of previous inflammatory coronary disease [2,3]. Kawasaki disease is of relevance because coronary involvement during childhood can result in late stenotic or occlusive coronary complications [4].

Electronic cigarette use has also become increasingly prevalent among young adults [5]. Although electronic cigarettes are frequently perceived as less harmful than combustible cigarettes, nicotine-containing electronic-cigarette aerosols have been associated with adverse cardiovascular effects [5,6]. However, as there are limited long-term studies on the contribution of electronic-cigarette exposure to clinical CAD, it is not possible to infer causality from individual cases [5,6].

We present a young adult man with exertional chest pain in whom coronary angiography revealed total occlusions of all three major coronary arteries. We describe the rationale for the subsequent aetiological investigations and highlight the importance of maintaining suspicion for CAD in young adults, particularly when the severity of disease is disproportionate to conventional cardiovascular risk factors.

## Case presentation

On Day 1, a 26-year-old man initially presented to a district general hospital with a three-day history of exertional chest pain, first noticed during a gym session. Episodes lasted two to three minutes and resolved with rest but recurred when exercise was resumed. He reported associated shortness of breath and palpitations but no nausea, vomiting, orthopnoea, paroxysmal nocturnal dyspnoea, or syncope. He recalled a coryzal illness with fever approximately three weeks prior to presentation.

His past medical history was unremarkable, with no previous surgery, regular or over-the-counter medications, or known allergies. There was no history of prolonged immobilisation, recent foreign travel, or long-haul travel. He was physically active and attended the gym three times per week. He drank alcohol only on special occasions and denied recreational or performance-enhancing drug use, including cocaine, amphetamines, and anabolic steroids. He reported a four-pack-year cigarette smoking history and had switched to electronic cigarettes three years earlier. He reported consuming approximately one disposable electronic-cigarette device per day, marketed as providing up to 2,500 puffs and labelled as containing 2% nicotine (20 mg/mL). There was no known family history of premature CAD or sudden cardiac death.

On admission, his blood pressure was 111/74 mmHg, pulse 64 beats per minute, respiratory rate 15 breaths per minute, temperature 36.2°C, and oxygen saturation 99% on room air. Cardiovascular and respiratory examinations were unremarkable, with a regular pulse, normal jugular venous pressure, no audible cardiac murmurs, clear lung fields and no peripheral oedema. There was no finger or toe clubbing and no evidence of xanthelasma. His body mass index of 22.39 kg/m^2^ (weight 65.9 kg; height 1.715 m) was in the healthy weight range.

The initial electrocardiogram (ECG) showed ST-segment elevation in leads V2-V4 and aVL. High-sensitivity troponin was elevated at 1,406 ng/L (reference range 0-14 ng/L). Chest radiography showed no cardiomegaly or pulmonary oedema. Bedside echocardiography demonstrated mildly impaired left ventricular systolic function with an estimated ejection fraction of 45% and inferoseptal hypokinesia. Capillary blood glucose and renal function were unremarkable. A non-fasting lipid profile was obtained (Table 1). Respiratory viral screening for influenza and COVID-19 was negative.

**Table 1:** Summary of routine haematological, biochemical, endocrine, viral, and lipid investigations. CRP: C-reactive protein; ALT: alanine aminotransferase; ALP: alkaline phosphatase; TSH: thyroid-stimulating hormone; FT4: free thyroxine; HbA1c: glycated haemoglobin; ESR: erythrocyte sedimentation rate; NT-proBNP: N-terminal pro-B-type natriuretic peptide; PCR: polymerase chain reaction; RSV: respiratory syncytial virus; SARS-CoV-2: severe acute respiratory syndrome coronavirus 2; HDL: high-density lipoprotein; LDL: low-density lipoprotein

Test	Value	Reference and Units
Haematology		
White cell count	7.2	4.0-11.0 x 10^9^/L
Red cell count	5.11	4.50-6.00 x 10^12^/L
Haemoglobin	149.0	130.0-180.0 g/L
Haematocrit	0.428	0.400-0.520 L/L
Mean cell volume	83.8	80.0-98.0 fL
Platelets	142	150-400 x 10^9^/L
Neutrophils	3.69	1.80-7.50 x 10^9^/L
Lymphocytes	2.35	1.00-4.00 x 10^9^/L
Monocytes	0.77	0.20-1.00 x 10^9^/L
Eosinophils	0.38	0.00-0.40 x 10^9^/L
Basophils	0.04	0.00-0.10 x 10^9^/L
Biochemistry		
Sodium	136	133-146 mmol/L
Potassium	4.3	3.5-5.3 mmol/L
Urea	5.7	2.5-7.8 mmol/L
Creatinine	86	59-104 µmol/L
eGFR	>90	>90 mL/min/1.73 m^2^
CRP	1	0-5 mg/L
Liver profile		
ALT	61	1-50 IU/L
ALP	74	30-130 U/L
Total bilirubin	10	0-21 µmol/L
Protein	76	60-80 g/L
Globulins	36	15-42 g/L
Bone profile		
Calcium	2.36	2.20-2.60 mmol/L
Adjusted calcium	2.37	2.20-2.60 mmol/L
Albumin	39	35-50 g/L
Phosphate	1.32	0.80-1.50 mmol/L
Endocrine and inflammatory markers		
TSH	2.03	0.27-4.20 mIU/L
FT4	17.6	11.3-21.6 pmol/L
HbA1c	36	20-41 mmol/mol
ESR	6	1-5 mm/hr
NT-proBNP	1433	0-400 ng/L
Respiratory viral screening		
Influenza A virus PCR	Negative	Negative
Influenza B virus PCR	Negative	Negative
Respiratory syncytial virus PCR	Negative	Negative
SARS-CoV-2 PCR	Negative	Negative
Non-fasting lipid profile		
Total cholesterol	2.0	<5.0 mmol/L
Triglycerides	0.6	0.0-1.6 mmol/L
HDL cholesterol	1.0	>1.0 mmol/L (male)
Direct LDL cholesterol	0.9	<3.0 mmol/L
Non-HDL cholesterol	1.0	<4.0 mmol/L
Total cholesterol to HDL ratio	2.0	<4
Additional lipid markers		
Lipoprotein (a)	<17	<32 nmol/L
Apolipoprotein B	0.36	<0.90 g/L

The patient was discussed with the tertiary cardiac centre regarding emergency transfer. Although he had ST-segment elevation and a markedly elevated high-sensitivity troponin concentration, he was haemodynamically stable and had no ongoing or resting chest pain at the time of assessment. His symptoms had been intermittent and exertional, with the first episode occurring more than 72 hours earlier. In the absence of ongoing ischaemic symptoms or clinical instability, immediate transfer for emergent invasive assessment was not pursued. He was treated locally for AMI. Treatment consisted of dual antiplatelet therapy with ticagrelor 180 mg and aspirin 300 mg, together with fondaparinux, atorvastatin 80 mg, ramipril 1.25 mg, bisoprolol 1.25 mg, and lansoprazole 15 mg. On Day 5, he was transferred to a tertiary cardiac centre for diagnostic coronary angiography.

On Day 5, high-sensitivity cardiac troponin had fallen to 64 ng/L (reference range 0-14 ng/L), and the ECG showed ST-segment elevation in leads V2-V4 and aVL, with T-wave inversion in leads III and aVF (Figure 1). The presenting ECG from Day 1 was not available for inclusion in this report; however, the contemporaneous clinical record documented ST-segment elevation in leads V2-V4 and aVL. Direct assessment of interval electrocardiographic changes was therefore not possible.

**Figure 1:**
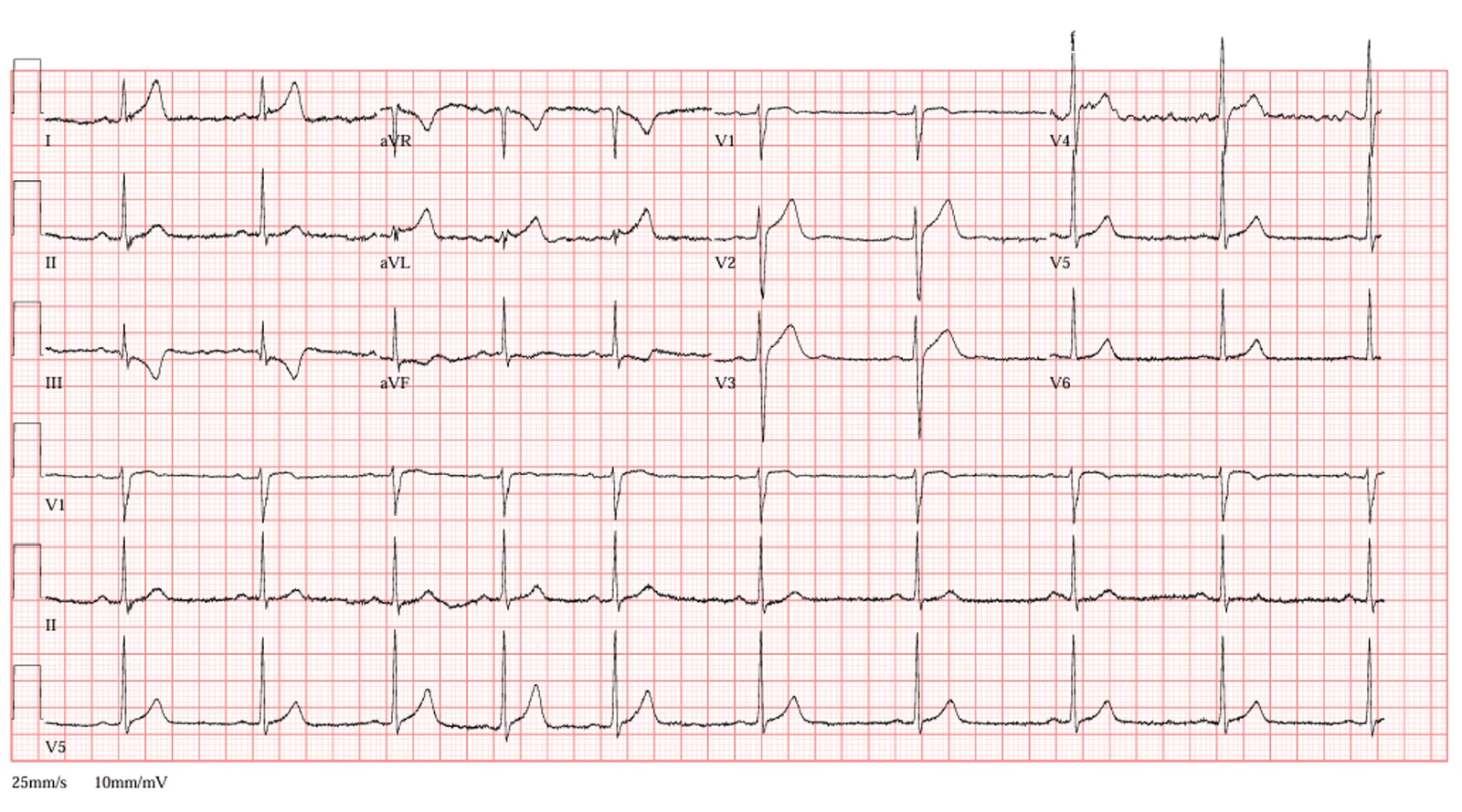
ECG taken on arrival at the tertiary cardiac centre. Note the ST-segment elevation in leads V2-V4 and aVL, with T-wave inversion in leads III and aVF.

Transthoracic echocardiography demonstrated normal left ventricular cavity dimensions and wall thickness, with global hypokinesia and mildly impaired left ventricular systolic function. The estimated left ventricular ejection fraction was 45%. Right ventricular size and systolic function were normal. Mild tricuspid and pulmonary valve regurgitation were present.

Coronary angiography was performed via the right radial artery and revealed severe three-vessel coronary disease. The left main stem was short without significant disease. There was complete occlusion of the mid left anterior descending artery (LAD), proximal left circumflex artery (LCx), and proximal right coronary artery (RCA) (Figures 2-4). These lesions were interpreted angiographically as chronic total occlusions, supported by collateral and retrograde filling of the distal vessels. However, the exact duration of each occlusion could not be established, and no definite angiographic evidence of superimposed acute thrombus was documented.

**Figure 2:**
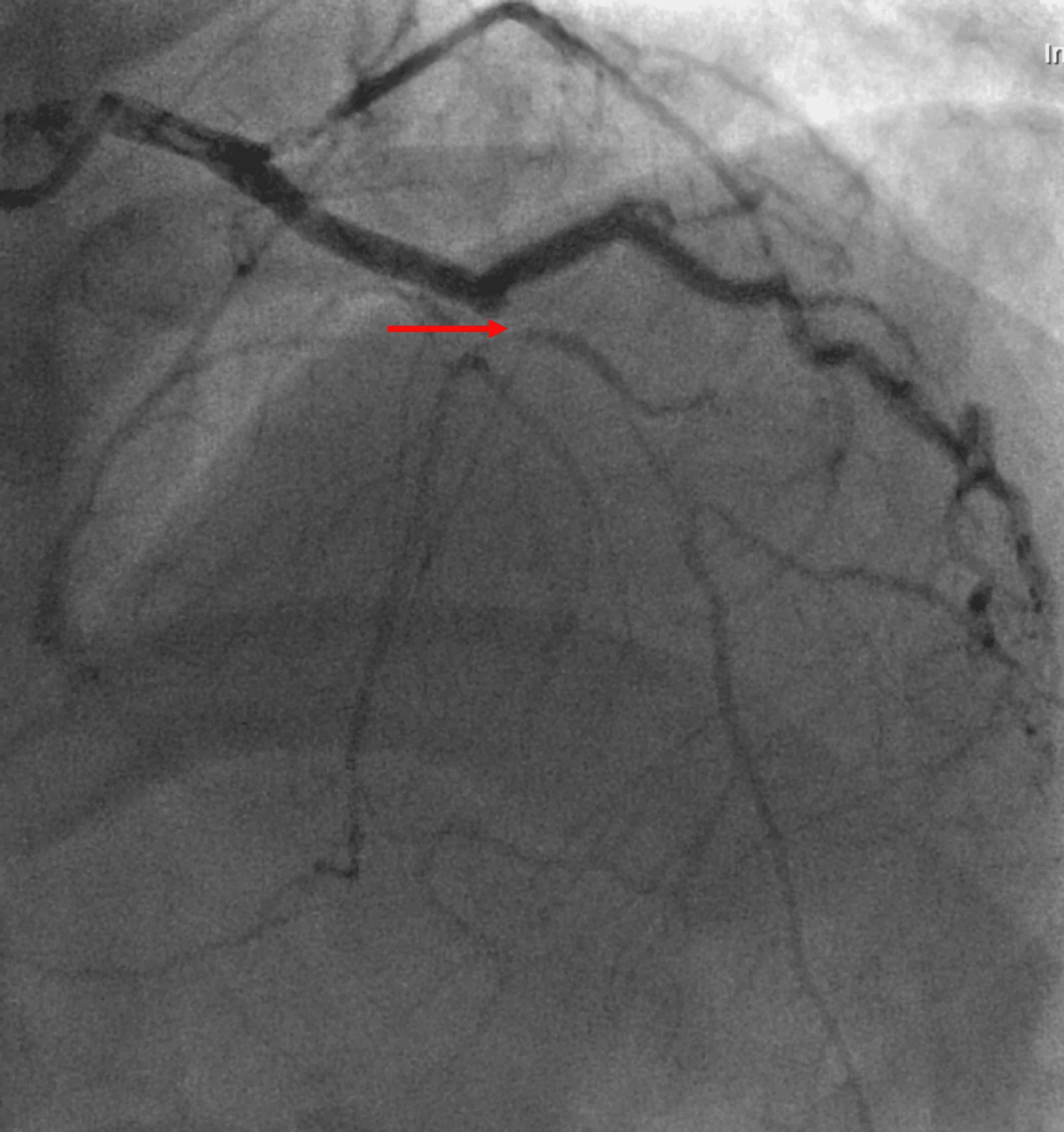
Left coronary angiography in posteroanterior cranial projection. The red arrow demonstrates an abrupt cut-off of the mid LAD, consistent with a total occlusion. LAD: left anterior descending

**Figure 3:**
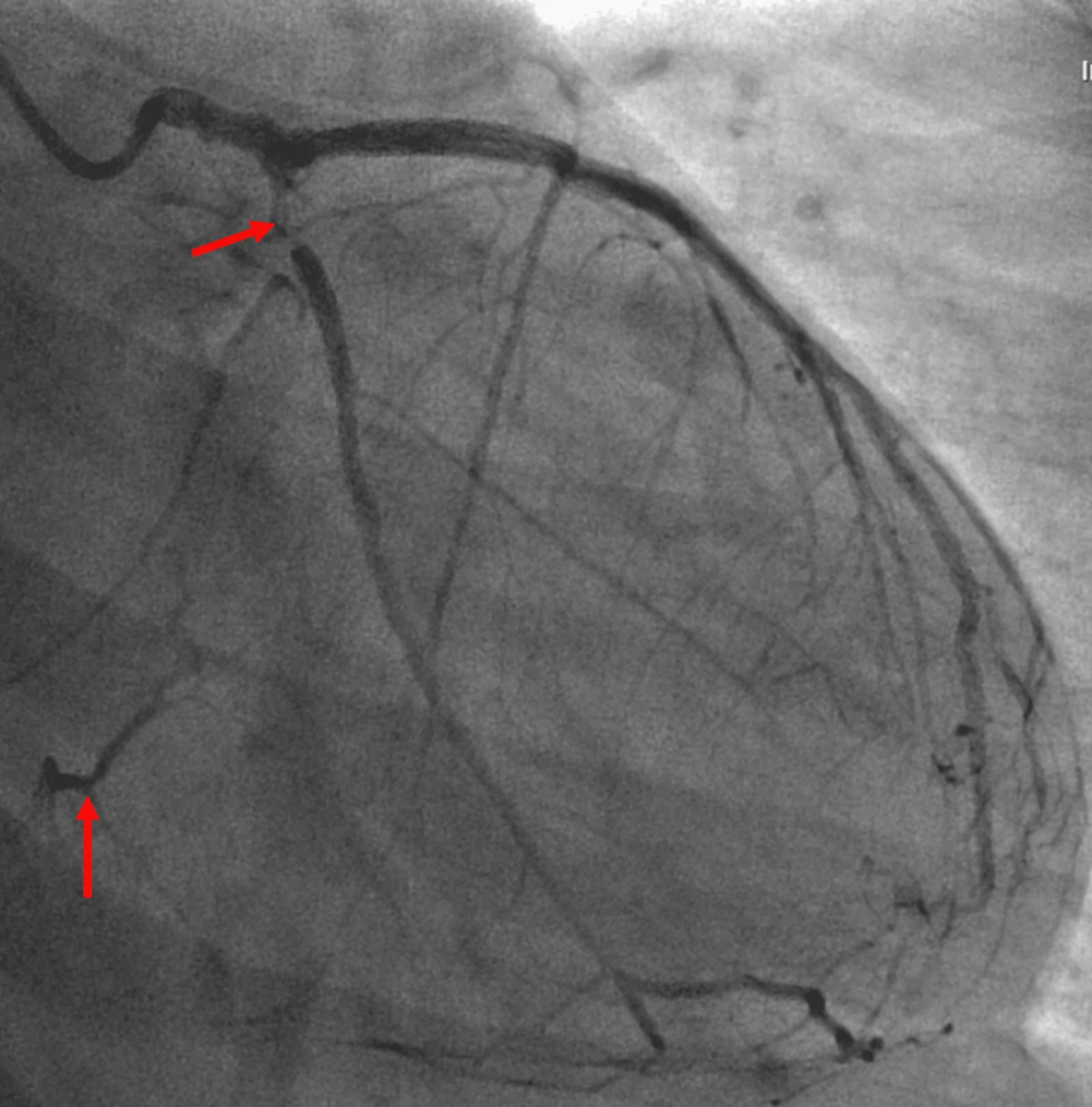
Left coronary angiography in right anterior oblique projection. The superior red arrow demonstrates abrupt occlusion of the proximal left circumflex artery in keeping with total occlusion. The inferior red arrow demonstrates retrograde opacification of the distal right coronary artery with left-to-right collateral filling.

**Figure 4:**
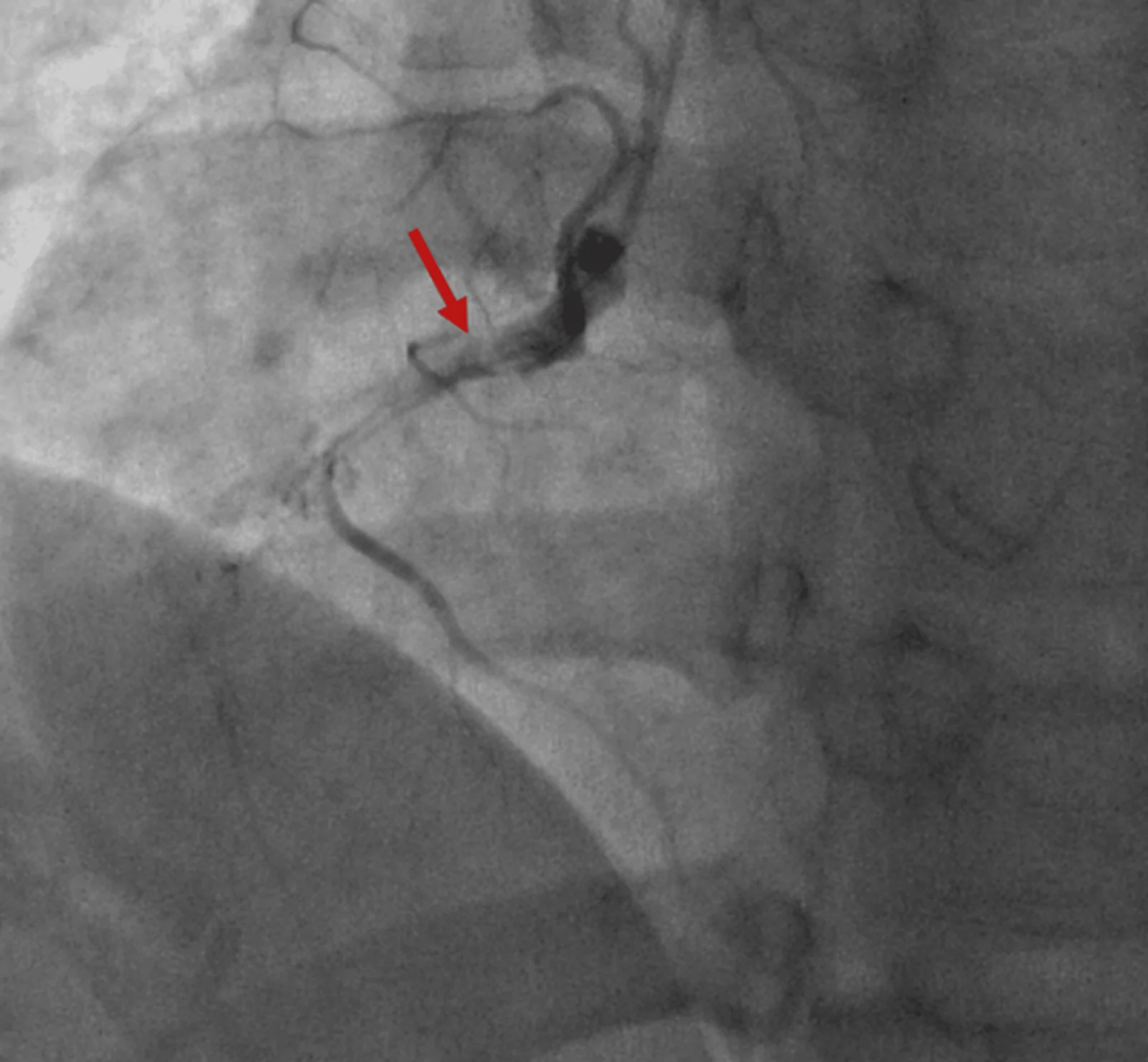
Right coronary angiography in anteroposterior caudal projection. The arrow shows opacification of a short proximal segment of the right coronary artery, followed by an abrupt cut-off, consistent with a total occlusion.

No definite infarct-related artery was identified angiographically. Complete occlusions were present in all three major coronary territories, with collateral and retrograde filling of the distal vessels, but the exact duration of each occlusion could not be established. There was no documented evidence of superimposed acute coronary thrombosis. In the context of severe multivessel disease with collateral-dependent perfusion, acute ischaemia within a markedly compromised coronary circulation may have contributed to the myocardial injury. However, the precise infarct-related territory and mechanism could not be established.

Blood investigations, including full blood count, renal function, liver function, C-reactive protein, thyroid function, lipid profile, glycated haemoglobin, bone profile and N-terminal pro-B-type natriuretic peptide were performed (Table 1).

The non-fasting lipid profile showed total cholesterol 2.0 mmol/L, low-density lipoprotein (LDL) cholesterol 0.9 mmol/L, high-density lipoprotein (HDL) cholesterol 1.0 mmol/L, and triglycerides 0.6 mmol/L. Glycated haemoglobin was 36 mmol/mol (reference <42 mmol/mol), providing no evidence of diabetes mellitus.

The extent of coronary disease, the patient’s young age and the absence of multiple conventional cardiovascular risk factors prompted additional investigations to identify an underlying aetiology. These included a detailed family pedigree, genetic testing for familial hypercholesterolaemia, lipoprotein(a) (Lp(a)), apolipoprotein B (apoB), plasma phytosterols, and investigations for inflammatory and autoimmune disease (Table 2).

**Table 2:** Summary of specialist metabolic, phytosterol, autoimmune, and vasculitis investigations. AI: antibody index; SS-A: Sjögren syndrome A; SS-B: Sjögren syndrome B; RNP: ribonucleoprotein; Sm: Smith; IgG: immunoglobulin G; dsDNA: double-stranded deoxyribonucleic acid; MPO: myeloperoxidase; PR3: proteinase 3; Scl-70: scleroderma-70; IgA: immunoglobulin A; IgM: immunoglobulin M

Test	Value	Reference and Units
Plasma phytosterol analysis		
Cholesterol (sterol analysis)	1610	2500-7200 µmol/L
7-Dehydrocholesterol	0.2	0-2.5 µmol/L
Lathosterol	0.3	0-12.3 µmol/L
Desmosterol	0.5	0-4.2 µmol/L
Sitosterol	5.3	0-8.7 µmol/L
Cholestanol	6.0	0-12.5 µmol/L
Campesterol	3.8	0-13.7 µmol/L
7-Dehydrochol/cholesterol ratio	0.12	0-0.40 µmol/mmol
Lathosterol/cholesterol ratio	0.19	0.25-2.65 µmol/mmol
Desmosterol/cholesterol ratio	0.31	0.14-0.75 µmol/mmol
Sitosterol/cholesterol ratio	3.29	0.21-1.9 µmol/mmol
Cholestanol/cholesterol ratio	3.73	1.00-2.70 µmol/mmol
Campesterol/cholesterol ratio	2.36	0.26-2.84 µmol/mmol
Autoimmune and vasculitis investigations		
Cardiolipin M	<1.5	0.0-19.9 MPLU
SS-A antibody	<0.2	0.0-0.9 AI
SS-A52 antibody	<0.2	0.0-0.9 AI
SS-A60 antibody	<0.2	0.0-0.9 AI
SS-B antibody	<0.2	0.0-0.9 AI
RNP 68	<0.2	0.0-0.9 AI
Anti-Sm antibody	0.3	0.0-0.9 AI
SmRNP antibody	1.2	0.0-0.9 AI
Ribosomal P	<0.2	0.0-0.9 AI
Chromatin	0.2	0.0-0.9 AI
Jo-1	<0.2	0.0-0.9 AI
Scl-70	0.4	0.0-1.6 AI
Antinuclear antibody (ANA)	Positive	Negative
Centromere	<0.2	0.0-0.9 AI
IgG dsDNA antibody	1.0	0.0-9.9 IU/mL
MPO	<0.2	0.0-0.9 AI
PR3	<0.2	0.0-0.9 AI
Immunofluorescent ANA	Speckled	Negative
Immunofluorescent ANA titre 1:300		
Rheumatoid factor	<10	0-13 kIU/L
IgG	15.80	6.00-16.00 g/L
IgA	1.92	0.80-2.80 g/L
IgM	0.59	0.50-1.90 g/L

Pedigree analysis confirmed that the patient had one older sister who was fit and well. Both his parents were alive and aged in their 60s, and he had no known family history suggestive of an inherited cardiovascular disorder. The patient was born in Nepal and moved to the United Kingdom aged 10 years. He denied known exposure to chemicals or pesticides but recalled a prolonged febrile illness lasting approximately one month when he was aged six and living in Nepal. He did not recall any associated rash or skin desquamation and attended hospital for assessment but did not require admission.

ApoB was 0.36 g/L (reference <0.90 g/L), and Lp(a) was <17 nmol/L (reference <32 nmol/L) (Table 1). Genetic testing for familial hypercholesterolaemia included analysis of APOB, APOE, LDLR, LDLRAP1, and PCSK9, but no pathogenic variant explaining the patient’s clinical presentation was identified. The precise timing of the lipid profile and ApoB measurements in relation to initiation of atorvastatin 80 mg could not be confirmed from the available records. These results were therefore interpreted cautiously, as both the AMI and high-intensity statin therapy may have influenced the measured lipid concentrations and could have resulted in underestimation of the untreated atherogenic lipoprotein burden.

Absolute sitosterol and campesterol concentrations were within their respective laboratory reference ranges, providing no biochemical evidence of sitosterolaemia (Table 2). Although the sitosterol-to-cholesterol and cholestanol-to-cholesterol ratios were elevated, these were interpreted in the context of the low cholesterol concentration and concurrent treatment with atorvastatin 80 mg. The abnormal ratios were therefore not considered diagnostic of sitosterolaemia in the absence of elevated absolute plant sterol concentrations, while suppression of cholesterol synthetic markers was considered consistent with statin therapy.

An 18F-fluorodeoxyglucose positron emission tomography (FDG-PET) scan was performed to investigate for inflammatory arteriopathy but found no abnormal FDG uptake within the large vessels of the thorax or abdomen and no metabolic evidence of active large-vessel vasculitis (Figure 5). Myeloperoxidase (MPO) and proteinase-3 (PR3) antibodies were negative, rheumatoid factor was <10 kIU/L, and complement levels were within their respective reference ranges. Antinuclear antibody (ANA) testing was weakly positive with a speckled immunofluorescence pattern and a reported titre of 1:300. Extractable nuclear antigen testing showed a mildly elevated SmRNP antibody of 1.2 AI (Table 2). Preoperative C-reactive protein was 1 mg/L, and his erythrocyte sedimentation rate was 6 mm/hour (Table 1).

**Figure 5:**
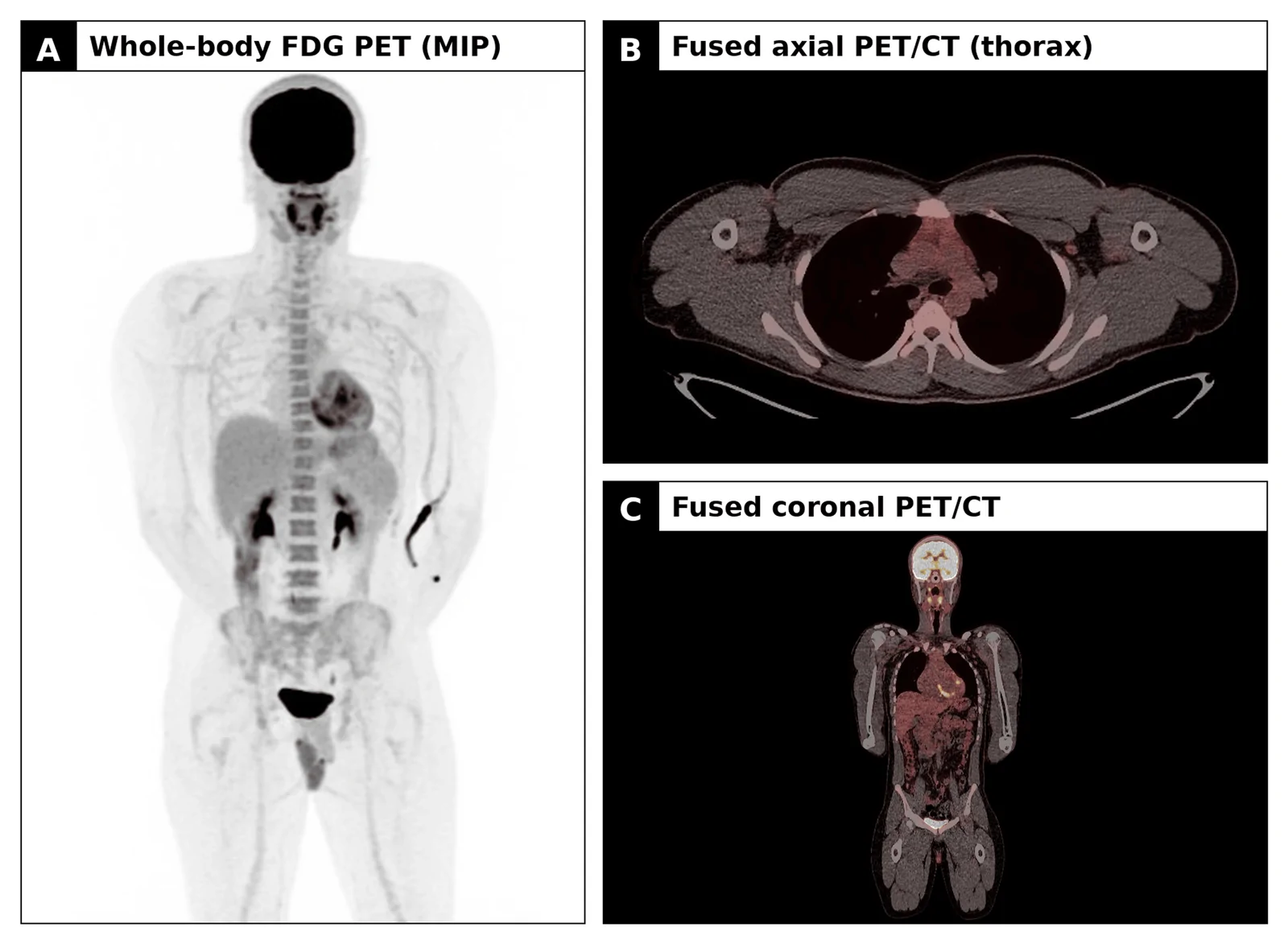
FDG PET-CT evaluation for large-vessel vasculitis. FDG PET-CT was performed to investigate possible large-vessel vasculitis in a young adult with severe premature multivessel coronary artery disease. Representative whole-body maximum intensity projection (A), fused axial thorax (B), and fused coronal PET/CT (C) images demonstrate no abnormal FDG uptake suggestive of active large-vessel vasculitis, in keeping with the original nuclear medicine report. FDG: fluorodeoxyglucose; MIP: maximum intensity projection; PET: positron emission tomography; CT: computed tomography

Following multidisciplinary heart team discussion, surgical coronary revascularisation was recommended. Preoperative investigations included carotid Dopplers, pulmonary function tests with transfer factor and a dental review.

The patient underwent coronary artery bypass grafting via median sternotomy. The left internal mammary artery was grafted to the obtuse marginal artery, a free right internal mammary artery graft was anastomosed to the distal LAD, and a saphenous vein graft was placed to the mid RCA. The available operative record did not document the specific anatomical or technical rationale for this conduit configuration. The left atrial appendage was also ligated and its tip excised, although the specific indication for this additional procedure was not documented and there was no known preoperative history of atrial fibrillation. The operating surgeon described the coronary arteries as having an arteritic-type appearance rather than the appearance of conventional atherosclerotic disease. This represented a subjective intraoperative impression; specific macroscopic features supporting this interpretation were not documented, and no histopathological confirmation was available. The available angiographic report did not describe aneurysmal change or other specific coronary abnormalities establishing an inflammatory arteriopathy. The intraoperative appearance therefore contributed to consideration of previous inflammatory coronary disease but was not diagnostic of coronary arteritis.

Postoperatively, the patient developed a left-sided pleural effusion that increased in size on thoracic ultrasound. Image-guided drainage was planned; however, the procedure was not performed after the patient developed presyncope. The effusion was subsequently managed conservatively with clinical and radiographic surveillance. Pre-discharge echocardiography demonstrated normal left ventricular size with a visually estimated ejection fraction of 50%. He was discharged with planned outpatient cardiology follow-up. A follow-up chest radiograph obtained two weeks after discharge demonstrated resolution of the pleural effusion, with minimal residual blunting of the left costophrenic angle (Figure 6). Detailed information regarding discharge cardiovascular medications, smoking or electronic-cigarette cessation counselling, and longer-term cardiology follow-up was not available at the time of manuscript preparation.

**Figure 6:**
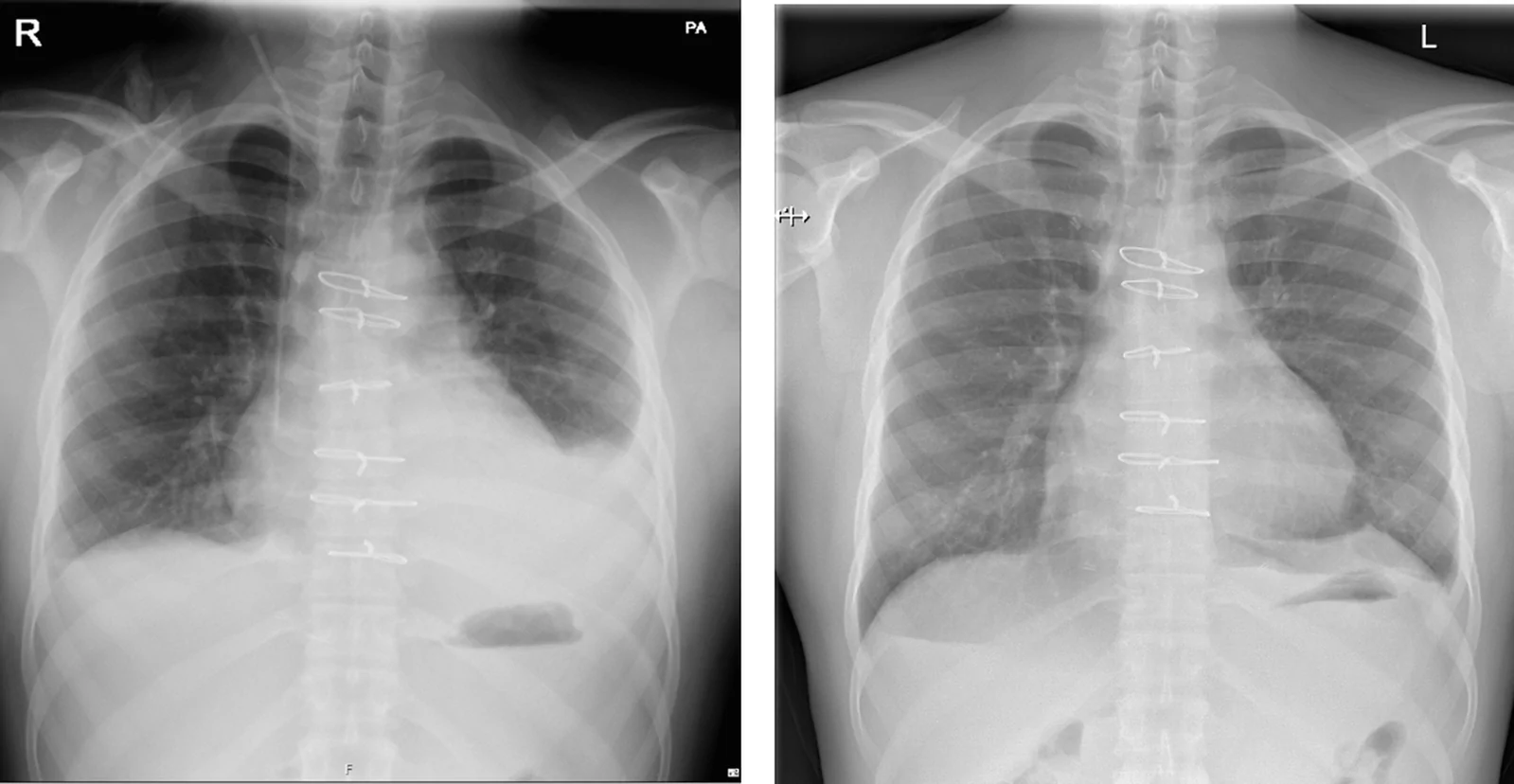
Postoperative left pleural effusion and subsequent radiographic resolution. Left panel: Chest radiograph demonstrating a moderate left-sided pleural effusion following coronary artery bypass grafting, with adjacent left basal atelectatic change. Right panel: Follow-up chest radiograph obtained two weeks after discharge showing resolution of the pleural effusion, with minimal residual blunting of the left costophrenic angle. Median sternotomy wires are visible on both radiographs.

## Discussion

This case demonstrates an unusual burden of obstructive coronary disease in a physically active 26-year-old man, with total occlusions involving all three major coronary territories. Although he had previous cigarette exposure and substantial ongoing electronic-cigarette use, he had no diabetes, hypertension, obesity or family history of premature CAD, and his measured lipid concentrations were not elevated. The severity of disease in the absence of multiple conventional risk factors prompted broader investigation for inherited, metabolic, inflammatory and autoimmune causes.

AMI is uncommon in young adults and encompasses a broad range of atherosclerotic and non-atherosclerotic mechanisms [7]. In patients younger than 30 years, reported cases occur predominantly in men and are often associated with conventional cardiovascular risk factors such as smoking and dyslipidaemia [1]. In this patient, myocarditis and pericarditis were initially considered because of his age and preceding viral-type illness. However, the exertional nature of his chest pain, markedly elevated troponin, ECG abnormalities and regional wall-motion abnormality provided objective evidence of myocardial injury requiring investigation for myocardial ischaemia. Although the clinical presentation was consistent with AMI, the relationship between the acute myocardial injury and the three total coronary occlusions remained uncertain. The lesions had been interpreted angiographically as chronic, with collateral and retrograde filling of the distal vessels; however, the exact duration of each occlusion could not be established, and no single infarct-related artery or superimposed acute thrombotic lesion was identified. The 2023 European Society of Cardiology guidelines emphasise assessment of suspected acute coronary syndrome using the overall clinical context, symptoms and early ECG findings rather than relying on age alone [8].

Atherosclerosis remains the most common cause of CAD in young adults, accounting for the majority of AMI cases in some series [2]. However, severe premature coronary disease should also prompt consideration of non-conventional contributors, including inflammatory and autoimmune conditions, inherited disorders and other acquired causes [2,3]. In this case, the extent of obstructive disease was disproportionate to the conventional cardiovascular risk profile, leading to assessment of inherited lipid disorders, inflammatory disease, unusual exposures and possible previous coronary inflammation.

Familial hypercholesterolaemia was considered because of its association with premature atherosclerotic cardiovascular disease. Genetic testing of LDLR, APOB, APOE, PCSK9 and LDLRAP1 did not identify a pathogenic variant explaining the phenotype. ApoB and LDL cholesterol were not elevated when measured; however, interpretation was limited by uncertainty regarding the timing of sampling in relation to initiation of high-intensity statin therapy and by the potential effects of the AMI on lipid concentrations. These measurements therefore could not definitively exclude a greater untreated atherogenic lipoprotein burden. ApoB remains an established marker of atherogenic particle burden [9]. Lp(a), an independent genetically determined cardiovascular risk factor, was within the laboratory reference range [10]. ESC guidance recommends Lp(a) measurement at least once in adulthood, particularly in individuals with premature atherosclerotic cardiovascular disease [11]. Sitosterolaemia was also considered because increased accumulation of plant sterols can cause accelerated atherosclerosis and premature CAD, but the absolute plant sterol concentrations provided no biochemical evidence of the condition [12].

Coronary vasculitis was another important consideration. Inflammation of the coronary vasculature may cause stenosis, thrombosis or aneurysm formation, particularly in conditions such as Kawasaki disease and polyarteritis nodosa [13]. The intraoperative description of an arteritic-type appearance increased the relevance of this possibility. However, FDG-PET showed no evidence of active large-vessel vasculitis, inflammatory markers were low, MPO- and PR3-specific ANCA were negative, and complement levels were normal. Although ANA was weakly positive and SmRNP mildly elevated, these findings were insufficient to establish systemic autoimmune disease. Importantly, the absence of active inflammation at the time of investigation does not exclude the possibility of remote inflammatory coronary injury.

The patient’s tobacco and electronic-cigarette exposure also required cautious interpretation. Cigarette smoking is a well-established cardiovascular risk factor associated with endothelial dysfunction, inflammation and oxidative stress [14,15]. Although his cumulative combustible-cigarette exposure was limited to four pack-years, he reported frequent electronic-cigarette use, consuming approximately one disposable device per day for three years, with each device marketed as providing up to 2,500 puffs. Evidence suggests that electronic-cigarette exposure may adversely affect cardiovascular physiology through mechanisms including endothelial dysfunction and oxidative stress [5,6]. However, long-term evidence directly linking electronic-cigarette use with severe premature obstructive coronary disease remains limited, and this case cannot establish causality.

Remote Kawasaki disease was also considered because of the patient’s history of a prolonged febrile illness during childhood. Coronary artery aneurysms may develop in untreated Kawasaki disease and can subsequently undergo remodelling, thrombosis, stenosis or occlusion, resulting in myocardial ischaemia later in life [4]. The combination of a prolonged childhood febrile illness, severe coronary disease at a young age and an intraoperative arteritic appearance raised this possibility. However, there was no documented history of other characteristic clinical features, childhood medical records were unavailable, and retrospective confirmation was not possible. Kawasaki disease therefore remained a plausible but unconfirmed explanation.

Despite extensive investigation, no definitive cause for the patient’s severe coronary disease was identified. This case highlights the importance of considering significant coronary pathology in young patients with compatible symptoms and objective evidence of myocardial injury, while also pursuing a structured investigation when the severity of disease is disproportionate to conventional cardiovascular risk factors. Equally, unusual exposures, serological abnormalities and historical findings should be interpreted cautiously and should not be assumed to establish causality in isolation.

## Conclusions

This case demonstrates that severe obstructive coronary disease can occur even in very young adults and should remain within the differential diagnosis when symptoms, electrocardiographic abnormalities and cardiac biomarkers suggest myocardial ischaemia. In young patients, alternative causes of chest pain may initially appear more likely; in this case, myocarditis and pericarditis were considered in view of his age and recent flu-like illness. However, his pain was not relieved by sitting forward, and the exertional nature of his symptoms raised concern for myocardial ischaemia.

The discovery of total occlusions involving all three major coronary territories at such a young age prompted extensive investigation for conventional, inherited, metabolic and inflammatory causes. Active systemic vasculitis, monogenic familial hypercholesterolaemia and biochemical evidence of sitosterolaemia were not demonstrated. However, the severity and unusual pattern of coronary disease, remote history of prolonged childhood febrile illness and intraoperative arteritic appearance raised the possibility of previous coronary inflammatory disease, including unrecognised Kawasaki disease. His exceptionally frequent electronic-cigarette exposure represented an additional potentially relevant cardiovascular exposure, although causality cannot be inferred from this case.
